# Supplementary figures and images for: Dysregulation of the DRAIC/SBK1 Axis Promotes Lung Cancer Progression
Source: Diagnostics (Basel). 2024 Oct 5;14(19):2227. doi: 10.3390/diagnostics14192227 (PMC11475998; doi:10.3390/diagnostics14192227)

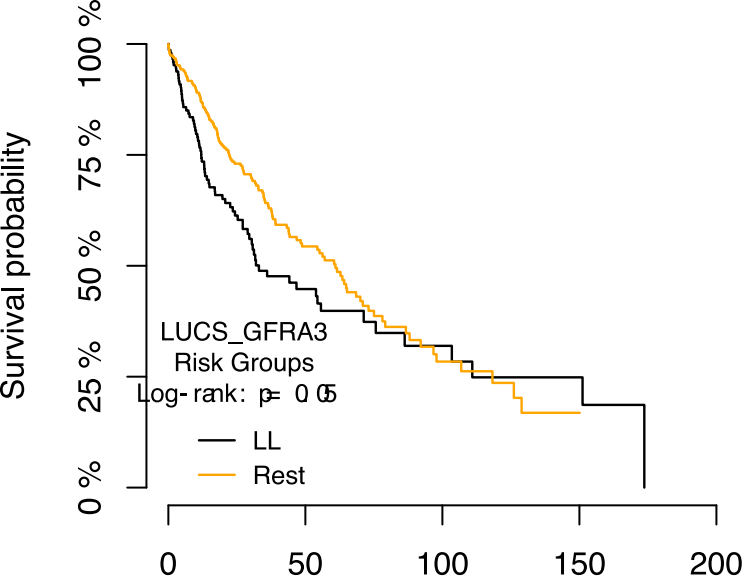

At Risk

|            |     |    |    |    |   |   |   |   |
|------------|-----|----|----|----|---|---|---|---|
| LL : 149   | 66  | 31 | 15 | 10 | 6 | 4 | 1 | 0 |
| Rest : 346 | 164 | 79 | 37 | 19 | 8 | 1 | 0 | 0 |

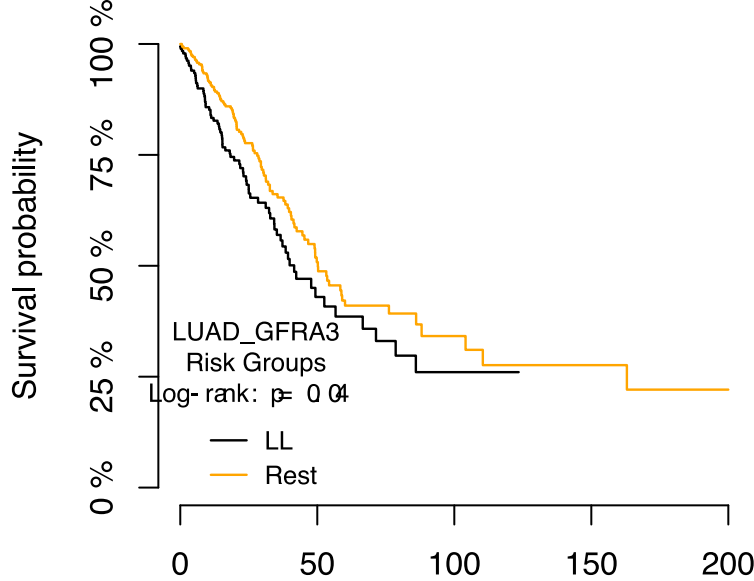

At Risk

|            |     |    |    |    |   |   |   |   |
|------------|-----|----|----|----|---|---|---|---|
| LL : 188   | 73  | 22 | 12 | 6  | 2 | 0 | 0 | 0 |
| Rest : 318 | 147 | 56 | 26 | 11 | 7 | 6 | 3 | 3 |

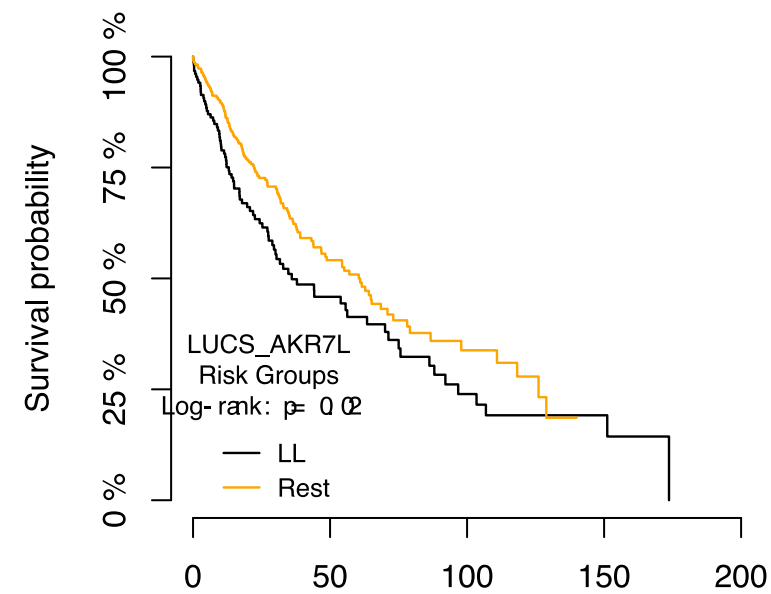

At Risk

|            |     |    |    |    |   |   |   |   |
|------------|-----|----|----|----|---|---|---|---|
| LL : 155   | 68  | 33 | 20 | 12 | 6 | 5 | 1 | 0 |
| Rest : 340 | 162 | 77 | 32 | 17 | 8 | 0 | 0 | 0 |

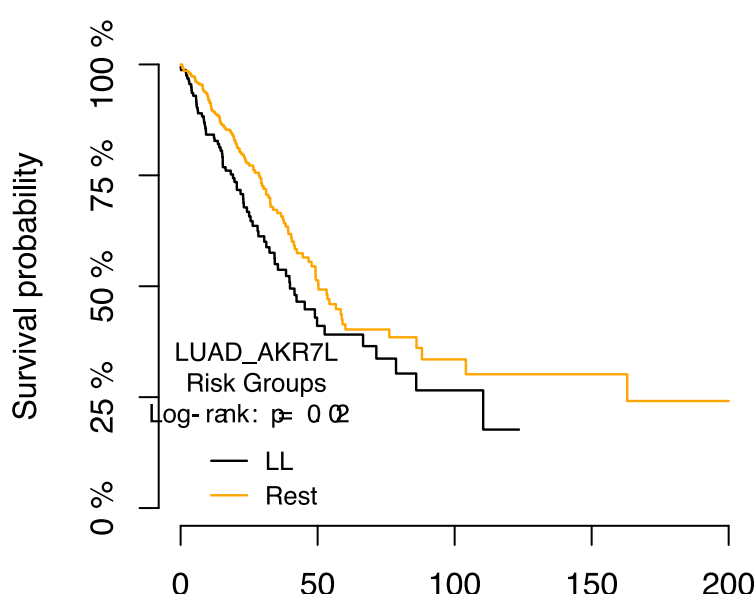

At Risk

|            |     |    |    |    |   |   |   |   |
|------------|-----|----|----|----|---|---|---|---|
| LL : 160   | 66  | 25 | 12 | 6  | 2 | 0 | 0 | 0 |
| Rest : 346 | 154 | 53 | 26 | 11 | 7 | 6 | 3 | 3 |

Supplement: Supplementary file 1 [file diagnostics-14-02227-s001.zip › S1.pdf]

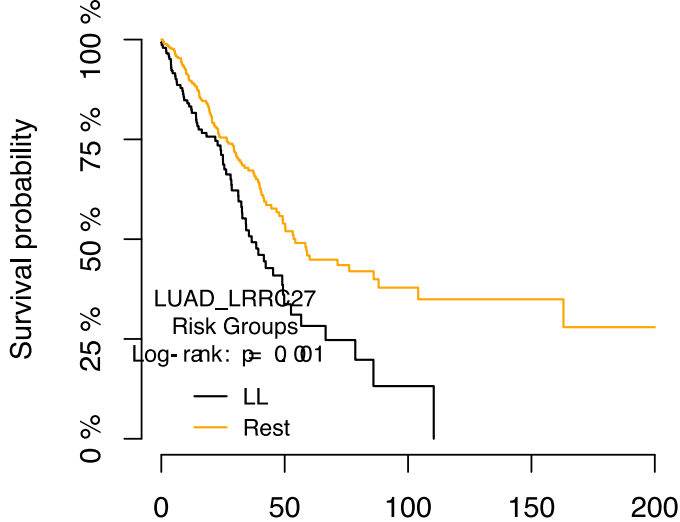

At Risk

|      |       |     |    |    |    |   |   |   |   |
|------|-------|-----|----|----|----|---|---|---|---|
| LL   | : 147 | 62  | 18 | 6  | 2  | 0 | 0 | 0 | 0 |
| Rest | : 359 | 158 | 60 | 32 | 15 | 9 | 6 | 3 | 3 |

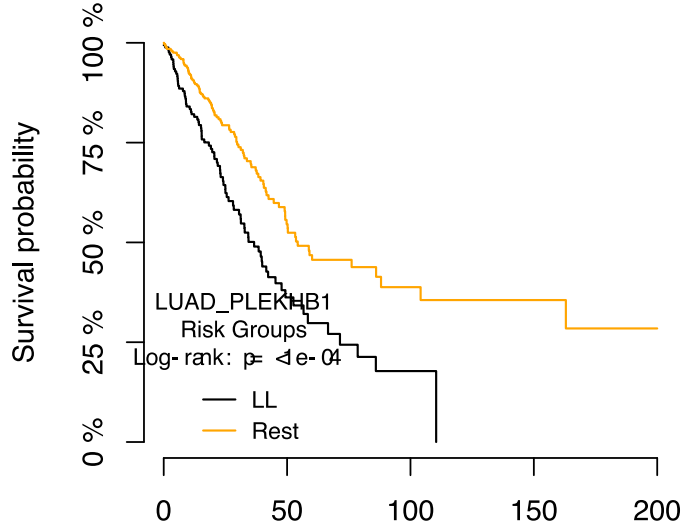

At Risk

|      |       |     |    |    |    |   |   |   |   |
|------|-------|-----|----|----|----|---|---|---|---|
| LL   | : 171 | 67  | 22 | 9  | 4  | 0 | 0 | 0 | 0 |
| Rest | : 335 | 153 | 56 | 29 | 13 | 9 | 6 | 3 | 3 |

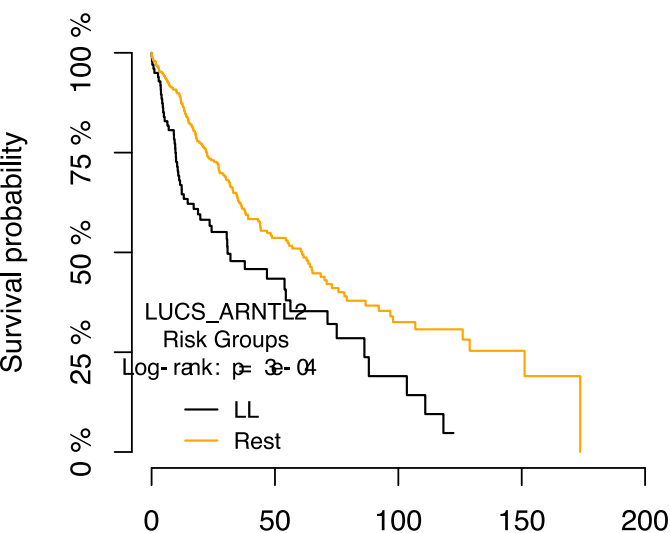

At Risk

|      |       |     |    |    |    |    |   |   |   |
|------|-------|-----|----|----|----|----|---|---|---|
| LL   | : 102 | 37  | 18 | 10 | 4  | 1  | 0 | 0 | 0 |
| Rest | : 393 | 193 | 92 | 42 | 25 | 13 | 5 | 1 | 0 |

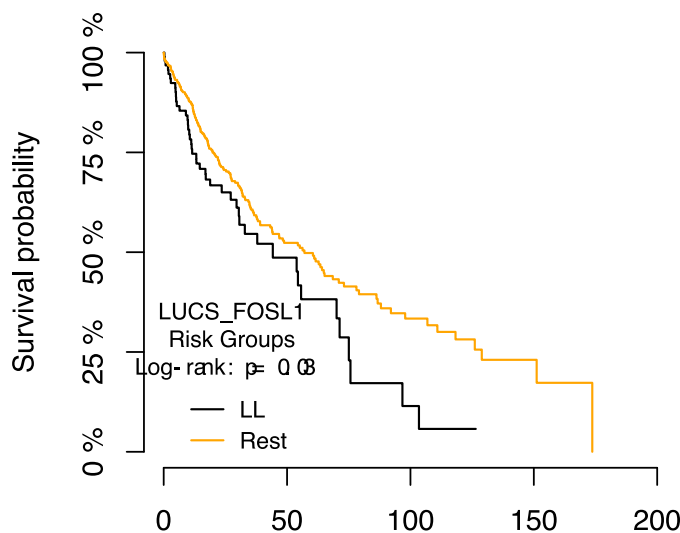

At Risk

|      |       |     |    |    |    |    |   |   |   |
|------|-------|-----|----|----|----|----|---|---|---|
| LL   | : 96  | 37  | 14 | 6  | 3  | 1  | 0 | 0 | 0 |
| Rest | : 399 | 193 | 96 | 46 | 26 | 13 | 5 | 1 | 0 |

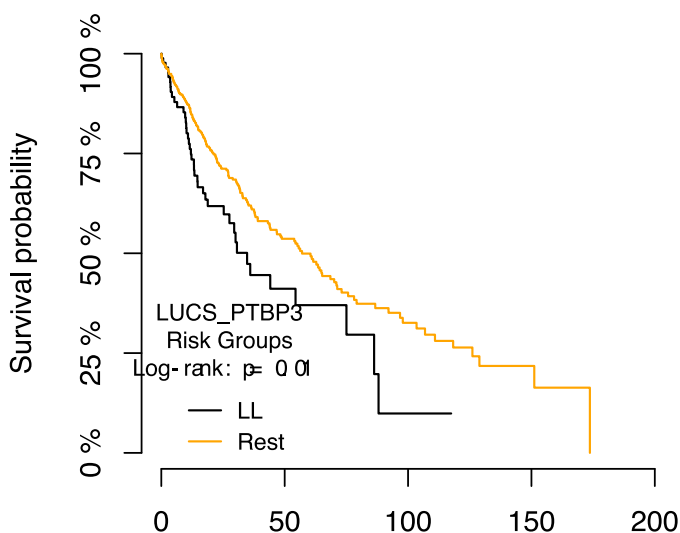

At Risk

|      |       |     |    |    |    |    |   |   |   |
|------|-------|-----|----|----|----|----|---|---|---|
| LL   | : 89  | 32  | 12 | 5  | 1  | 0  | 0 | 0 | 0 |
| Rest | : 406 | 198 | 98 | 47 | 28 | 14 | 5 | 1 | 0 |

Supplement: Supplementary file 1 [file diagnostics-14-02227-s001.zip › S2.pdf]
